# Cadmium Induces p53-Dependent Apoptosis in Human Prostate Epithelial Cells

**DOI:** 10.1371/journal.pone.0033647

**Published:** 2012-03-20

**Authors:** Pierpaolo Aimola, Marco Carmignani, Anna Rita Volpe, Altomare Di Benedetto, Luigi Claudio, Michael P. Waalkes, Adrie van Bokhoven, Erik J. Tokar, Pier Paolo Claudio

**Affiliations:** 1 Department of Biochemistry and Microbiology, Joan C. Edwards School of Medicine, Marshall University, Huntington, West Virginia, United States of America; 2 Department of Basic and Applied Biology, University of L'Aquila, L'Aquila, Italy; 3 Department of Urology, National Cancer Institute “Fondazione Senatore Pascale”, Naples, Italy; 4 National Toxicology Program at the National Institute of Environmental Health Sciences, Research Triangle Park, North Carolina, United States of America; 5 Department of Pathology, University of Colorado Denver, Aurora, Colorado, United States of America; 6 Department of Surgery, Joan C. Edwards School of Medicine, Marshall University, Huntington, West Virginia, United States of America; Harvard Medical School, United States of America

## Abstract

Cadmium, a widespread toxic pollutant of occupational and environmental concern, is a known human carcinogen. The prostate is a potential target for cadmium carcinogenesis, although the underlying mechanisms are still unclear. Furthermore, cadmium may induce cell death by apoptosis in various cell types, and it has been hypothesized that a key factor in cadmium-induced malignant transformation is acquisition of apoptotic resistance. We investigated the *in vitro* effects produced by cadmium exposure in normal or tumor cells derived from human prostate epithelium, including RWPE-1 and its cadmium-transformed derivative CTPE, the primary adenocarcinoma 22Rv1 and CWR-R1 cells and LNCaP, PC-3 and DU145 metastatic cancer cell lines. Cells were treated for 24 hours with different concentrations of CdCl_2_ and apoptosis, cell cycle distribution and expression of tumor suppressor proteins were analyzed. Subsequently, cellular response to cadmium was evaluated after siRNA-mediated p53 silencing in wild type p53-expressing RWPE-1 and LNCaP cells, and after adenoviral p53 overexpression in p53-deficient DU145 and PC-3 cell lines. The cell lines exhibited different sensitivity to cadmium, and 24-hour exposure to different CdCl_2_ concentrations induced dose- and cell type-dependent apoptotic response and inhibition of cell proliferation that correlated with accumulation of functional p53 and overexpression of p21 in wild type p53-expressing cell lines. On the other hand, p53 silencing was able to suppress cadmium-induced apoptosis. Our results demonstrate that cadmium can induce p53-dependent apoptosis in human prostate epithelial cells and suggest p53 mutation as a possible contributing factor for the acquisition of apoptotic resistance in cadmium prostatic carcinogenesis.

## Introduction

Prostate cancer is a major cause of morbidity and mortality in humans. With an estimated 240,890 new cases, (accounting for 29% of all expected male cancers) and 33,720 deaths (11% of total deaths caused by cancer in men) in 2011 in the USA, it is currently the most commonly diagnosed cancer and the second leading cause of cancer-related deaths in American men [1]. In spite of the continuous research advance on understanding molecular mechanisms and pathways involved in prostate cancer growth and progression, particularly regarding the role of androgen signaling [2], we are still far from clearly defining the complex etiology of this disease, which probably includes genetic background, age and physiologic status, lifestyle (e.g. diet and tobacco smoking) and exposure to other environmental risk factors.

In the vast group of environmental pollutants, the toxic heavy metal cadmium is considered a likely candidate as a causative agent for prostate cancer. Widely distributed and used in industry, and with a broad range of target organs and a long half-life (10–30 years) in the human body, this element has been long known for its multiple adverse effects on human health, through occupational or environmental exposure [3]. Moreover, cadmium and cadmium compounds have been classified by the International Agency for Research on Cancer and the U. S. National Toxicology Program as Group 1 human carcinogens, mainly on the basis of epidemiological studies showing a dose-response relationship between the level of cadmium exposure and the incidence of lung cancer in the human population [4], [5], while different experimental animal studies have clearly demonstrated that this metal can induce tumor formation at multiple tissue sites in various species [3]. The role of cadmium as a prostate carcinogen has been suggested by epidemiological data and results of several animal and *in vitro* studies. Some evidence exists that environmental cadmium exposure might be associated with prostate cancer in humans [5], [6]. And several studies demonstrated that cadmium induces tumors (adenocarcinomas) and preneoplastic (hyperplastic) lesions of the prostate in rats [4], [5], [7]–[10]. On the other hand, some authors reported evidence of *in vitro* malignant transformation of cultured prostate epithelial cells by cadmium, both with murine [11] and human cells [12], [13].

Nevertheless, the specific molecular events associated with cadmium-induced transformation are still elusive. Actually, considering the multiple molecular targets that have been identified, this metal probably operates with a complex oncogenetic mechanism, in which more than a single pathway could be implicated [14]. Cadmium can affect cell proliferation and differentiation, cell cycle progression, DNA synthesis and repair, apoptosis and other cellular activities [15], [16]. On the basis of currently available data, cadmium seems to have little direct genotoxic activity at doses relevant for potential cell transformation [6]. However, apparently it can inhibit DNA repair [17], [18], and thus represent a cause of genomic instability, a condition that has been associated with tumorigenesis, and even act as a co-genotoxic chemical [19]. Different other possible indirect mechanisms have also been proposed to explain cadmium carcinogenesis, including oxidative stress, proto-oncogene activation, altered DNA methylation and dysregulated gene expression [14]–[16]. In addition, a critical role has been proposed for acquisition of cellular apoptotic resistance following exposure to cadmium. As a fundamental defense mechanism against the uncontrolled proliferation of mutated or transformed cells in the body, apoptosis that is frequently observed in cadmium-exposed cells would be expected to have an anti-carcinogenic function. But in this context cadmium-induced apoptotic cell death might result not completely protective against malignant transformation, as suggested by some studies showing that only a fraction of exposed cells in a population die by apoptosis, while the rest may become apoptosis-resistant [19], [20]. Furthermore, it has been shown that cadmium-transformed or -adapted cells are characterized by increased resistance to apoptosis [21], [22], which may render them more prone to accumulation of mutations and neoplastic transformation. Indeed, disruption of apoptosis is considered critical in tumor formation and malignant progression, and acquired resistance to apoptosis is a general hallmark of cancer [23]. Therefore, an accurate characterization of the cellular and molecular response of prostate cells to cadmium cytotoxic action, particularly in terms of apoptosis induction, would be of the greatest interest. On account of this, and with the aim of giving further experimental contribution to this matter, we carried out an investigation on the effects of cadmium exposure on apoptosis and the expression of some tumor suppressor proteins in cultured cells derived from human prostate epithelium. In particular, the normal RWPE-1 cell line was tested and confronted with the response of its cadmium-transformed derivative CTPE. Subsequently, different prostate cancer cell lines were analyzed, including primary adenocarcinoma (22Rv1 and CWR-R1) and metastatic adenocarcinoma cells (LNCaP, PC-3 and DU145).

## Materials and Methods

### Cell lines, culture conditions and treatment with cadmium

Seven different human prostate epithelial cell lines were used (Table 1). RWPE-1 is a HPV18 immortalized, non-tumorigenic prostatic cell line which was established in 1997 from a histologically normal prostate [24]. On the other hand, the cadmium-transformed CTPE cell line was derived from RWPE-1 cells that acquired features of malignant nature after chronic exposure to 10 µM cadmium chloride [12]. 22Rv1 and CWR-R1 are related cell lines, both derived from a subline (CWR22R-2152) established from a xenograft of the primary tumor CWR22R [25]. LNCaP, DU145 and PC-3 are classical metastatic prostate adenocarcinoma cell lines, derived, respectively, from lymph node, brain and bone metastasis. All cell lines were maintained at 37°C in a humidified atmosphere containing 5% CO_2_ and passed weekly. RWPE-1 and CTPE cells were grown in Keratinocyte Serum-Free Medium (K-SFM) containing 50 µg/ml bovine pituitary extract (BPE), 5 ng/ml human (recombinant) epidermal growth factor (EGF) and 1% penicillin/streptomycin antibiotic solution. The other cell lines (22Rv1, CWR-R1, LNCaP, DU145 and PC-3) were grown in RPMI 1640 medium supplemented with 10% FBS. K-SFM and supplements (BPE and EGF) were purchased from Gibco, USA, while RPMI 1640 and FBS were obtained from Invitrogen Life Technologies, USA.

**Table 1:** Origin of the human prostate epithelial cell lines included in this study and mutation analysis of their ***p53*** locus.

Cell line	Origin	Ref.	*p53* Exon	Nucleotide change	Codon change	Ref.
**RWPE-1**	Normal prostate^a^	[24]		Wild type		[24]
**CTPE**	RWPE-1 cells^b^	[12]		Wild type		[12]
**22Rv1**	Primary tumor xenograft^c^	[29]	9	CAG→CGG	Q331R	[25]
**CWR-R1**	Primary tumor xenograft^c^	[25], [30]	8/9	CGT→CAT/CAG→CGG	A273H/Q331R	[25]
**LNCaP**	Lymphnode metastasis	[28]		Wild type		[34], [35]
**DU 145** d	Brain metastasis	[31], [32]	6/8	CCT→CTT/GTT→TTT	P223L/V274F	[34], [35]
**PC3**	Bone metastasis	[33]	5	GCC→GC (del. C)	138, frameshift, STOP codon 169	[34], [35]

a HPV18 immortalized cells.

b Transformed by chronic CdCl_2_ exposure.

c Subline CWR22R-2152.

d Both alleles mutated, mutations in exons 6 and 8 in different alleles [53].

For short-term treatment with cadmium, cells were maintained in culture medium containing cadmium chloride, at a desired concentration, for 24 h.

### Flow cytometric analysis of cell cycle distribution

To evaluate the percentages of cells at the various stages of the cell cycle, flow cytometric analysis of nuclear DNA content was performed. Following treatment, cells were harvested with trypsin, washed with PBS, fixed in cold (−20°C) 70% ethanol (about 10^6^ cells in 10 ml) and then stored in 70% ethanol at +4°C. Immediately before FACS analysis, fixed cells were washed with PBS, resuspended in DNA staining solution (20 µg/ml propidium iodide and 25 µg/ml RNase A (Sigma) in PBS), and incubated at 37°C for 30 min. Subsequently, cell cycle distribution (DNA histograms) was analyzed by using a BD FACS Aria flow cytometer and the BD FACS Diva software (v5.0.1). Cell doublets and cell aggregates were not gated during analyses.

### Flow cytometric detemination of apoptosis

Apoptotic cell death in prostate cell line cultures was determined using an Annexin-V FITC Apoptosis Detection kit (Bender MedSystems, GmbH, Vienna, Austria) according to the manufacturer's instructions. Briefly, cells were harvested, washed with PBS and resuspended in Annexin-V binding buffer. Then, the cells were stained with FITC-conjugated Annexin-V (1∶40 diluted stock solution in binding buffer) for 10 min at room temperature in the dark. After washing with binding buffer and staining with propidium iodide (1 µg/ml), cells were finally analyzed on a BD FACS Aria flow cytometer using BD FACS Diva software (v5.0.1). Early apoptotic cells were identified as Annexin V-positive/propidium iodide-negative cells.

### p53-specific siRNA transfection

For p53 silencing, RWPE-1 and LNCaP cells were seeded in 6-well plates at a density of 1×10^5^ cells per well, grown up to 40–50% confluence and then transfected with p53-specific siRNA (Santa Cruz Biotechnology Inc., Santa Cruz, CA), according to manufacturer's protocols. Briefly, 8 µl of p53 siRNA stock solution (10 µM) and 6 µl of transfection reagent (Santa Cruz Biotechnology Inc.) were separately diluted in 100 µl transfection medium (Santa Cruz Biotechnology Inc.) each, and incubated for 30 min at room temperature; then they were mixed together and further diluted with transfection medium up to a final volume of 1 ml (final concentration of p53 siRNA = 40 nM). After washing with transfection medium, cells were incubated with the mixture for 6 hours (100 µl per well), then an equal volume of culture medium with double concentrated-supplements was added and the cells incubated for additional 18 hours. At 24 hour post-transfection the transfection mixture was replaced with fresh complete medium and the cells were cultured for 24 hours before experiments of cadmium chloride exposure. For both RWPE-1 and LNCaP cell lines a FITC-conjugated unspecific siRNA (Santa Cruz Biotechnology Inc.) was used as a control for siRNA experiments, and optimal conditions for transfection (siRNA and transfection reagent concentrations, incubation times) were determined in preliminary experiments by using fluorescence microscopy evaluation of the transfection efficiency.

### Adenovirus infection (Adp53-mediated gene transfer)

A recombinant E1-deleted replication-defective adenoviral vector encoding human wild type p53 (Adp53), with the *p53* gene under the control of the human cytomegalovirus enhancer/promoter, was generated using the AdEasy system (Carlsbad, CA). Viral stock was prepared from a single clone of the Adp53 virus, obtained by plaque purification and amplified in the HEK 293 cell line (ATCC CRL-1573), carrying, and stably expressing, the E1 gene required for adenoviral replication.

For transduction of wild-type p53-deficient prostate cell lines, DU145 and PC-3 cells were seeded in 10 cm plates and cultured up to 40–50% confluence. Viral stock was diluted in RPMI 1640 supplemented with 2% heat-inactivated FBS and added directly to monolayer cultures. To avoid cytotoxic effects on target cells, preliminary tests were performed by using increasing MOI (Multiplicity Of Infection) of Adp53 for each cell line. Subsequently, cells were infected with 50 MOI of Adp53. After 8 h incubation, medium was replaced with complete 10% FBS RPMI 1640 and cells were grown for additional 16–24 hours before starting treatment with CdCl_2_.

### Western blot analysis

After the indicated treatments, cells were harvested and washed twice with cold PBS, and the resulting dry cell pellets were immediately stored at −80°C. For total cellular protein extraction, cells were lysed in ice-cold lysis buffer (50 mM Tris-HCl, pH 7.4, 250 mM NaCl, 50 mM NaF, 5 mM EDTA, 0.1% Triton X-100, 0.1 mM Na_3_VO_4_) containing protease inhibitors (Complete, Mini, EDTA-free from Roche), for 30 min at 4°C. The lysates were cleared by centrifugation at 14,000 g for 10 min at 4°C and the supernatants were either used immediately or stored at −80°C until use. Protein concentrations were determined by using the ND-1000 Spectrophotometer (Agilent Technologies). For immunoblot analysis, equal amounts of protein (30–50 µg) were mixed with loading buffer and boiled for 5 min. The samples were then loaded and resolved on 7–12% SDS-polyacrylamide gels. After electrophoresis, the proteins were electrotransferred to nitrocellulose membranes. The blots containing the transferred proteins were blocked with 5% non-fat dry milk in TBS-T (20 mM Tris-HCl, pH 7.6, 137 mM NaCl, 0.5% Tween 20), for 1 hour at room temperature or overnight at 4°C, and then incubated with primary antibodies in TBS-T containing 3% non-fat dry milk for 2 hours at room temperature or overnight at 4°C. The following primary antibodies were used: mouse monoclonal antibodies against p53 (1∶500), p21 (1∶500), β-actin (1∶5,000), and nucleolin C-23 (1∶1,000) (Santa Cruz Biotechnology, Santa Cruz, CA); rabbit polyclonal antibodies against p27 (1∶500), p16 (1∶250), pRb/p105 (1∶1,000), p107 (1∶250), and pRb2/p130 (1∶250) (Santa Cruz Biotechnology, Santa Cruz, CA); and a mouse monoclonal antibody against Glyceraldehyde-3-phosphate dehydrogenase/GAPDH (1∶1,000) (Chemicon, Billerica, MA). After washing, membranes were finally incubated with appropriate, species-specific horseradish peroxidase-conjugated secondary antibodies, used at 1∶10,000 or 1∶20,000 dilution in TBS-T for 1 hour at room temperature. Immunocomplexes were detected with the enhanced chemiluminescence system (ECL, Amersham, CA) and by autoradiography using X-ray film (FUJI Photo Film Co, Inc., Tokyo, Japan). Protein expression levels were determined semi-quantitatively by densitometric analysis with the Quantity One software (BioRad) using scanned autoradiographic images.

### Statistical analysis

Data/results are presented as means ± SD or SEM, calculated from at least three replicate determinations of the endpoints examined. The significance of the differences between treatments and respective controls was determined by Student's *t* test. *P*<0.05 was considered statistically significant (* *P*<0.05; ** *P*<0.01; *** *P*<0.001).

## Results

### Apoptosis induction by cadmium in RWPE-1 and CTPE cells

**Figure 1:**
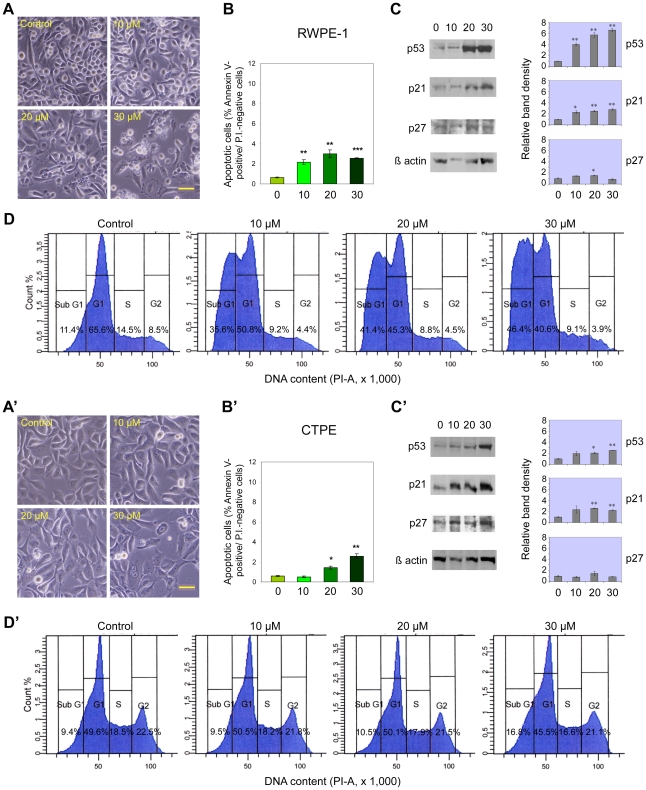
Cadmium induces dose-dependent apoptosis and p53 overexpression in RWPE-1 and CTPE human prostate cells. Cadmium effects on RWPE-1 (A–D) and CTPE (A′–D′) cells, after 24-h treatment with different concentrations of CdCl_2_ (0, 10, 20 and 30 µM). A, A′: representative phase contrast microscopy images. B, B′: early apoptosis detection by FITC-conjugated Annexin-V/PI and FACS analysis. C, C′: western blot analysis of p53, p21 and p27 expression. D, D′: FACS analysis of cell DNA content. Histograms in B, B′ report mean percentages ± SEM of Annexin-V positive/PI-negative cells (n = 3). Histograms in C, C′ represent relative band densities (mean ± SEM, n = 3), as determined by densitometry, using β-actin as the loading control for standardization. Scale bars in A, A′ = 100 µm. * P<0.05, ** P<0.01, *** P<0.001.

To evaluate the ability of cadmium to trigger apoptotic cell death in prostate epithelial cells at concentrations comparable with observed *in vivo* levels in exposed humans, the cells were treated for 24 hours with different concentrations of cadmium chloride, in the range 0–30 µM, and early apoptosis was detected and quantified by means of FACS analysis after staining with FITC-conjugated Annexin-V and propidium iodide, while parallel FACS analysis of cell DNA content was used to assess the cell cycle distribution. Cadmium exposure was clearly responsible for the induction of apoptosis in RWPE-1 normal prostate cells (Fig. 1B), as evidenced by a dose-dependent increase in the frequency of early apoptotic cells (Annexin V-positive/propidium iodide-negative). This effect was already obtained at 10 µM cadmium chloride (about 3.5-fold increase, p<0.01), and could be easily observed under the inverted microscope, with apoptotic cells exhibiting cytoplasmic shrinkage and/or blebbing, and detaching from each other or floating in the medium (Fig. 1A). By FACS analysis of cell DNA content, a remarkable increase in sub-G1 fraction was also seen (Fig. 1D). Since sub-G1 peaks include early and late apoptotic cells, but also a part of necrotic cells, such large sub-G1 fractions provide strong evidence for cadmium cytotoxicity resulting in a global reduction in the number of viable cells, which is also reflected in decreased S+G2/M fractions. CTPE cells (cadmium-transformed derivatives of RWPE-1) resulted more resistant to apoptosis induction, which was observed only at concentrations = 20 µM (about 2.5-fold increase, p<0.05; Fig. 1A′,B′). Moreover, clear effects on cell cycle distribution, with some increase in sub-G1 fraction, were obtained only at the highest concentration employed (30 µM; Fig. 1D′).

### Western blot analysis of p53, p21 and p27 tumor suppressor protein expression in RWPE-1 and CTPE cells

To shed light onto the molecular mechanisms underlying the response of prostate epithelial cells to cadmium cytotoxic activity, and to assess the possible involvement of tumor suppressor proteins that are frequently altered or deregulated in most types of cancer, western blot analysis was used to detect the levels of total p53, p21 and p27 proteins in RWPE-1 and CTPE cells (Fig. 1C, C′). Cadmium treatment resulted in significant concentration-dependent elevations of total p53 in RWPE-1 and, to a lesser extent, CTPE cells. A parallel dose-dependent increase was also observed in the levels of p21, a protein that is produced by a target gene of p53. As for p27, no significant variations in the levels of this protein were observed after 24-hour cadmium treatment in both cell lines, apart from a modest, 1.5-fold increment at 20 µM cadmium chloride in RWPE-1.

Parallel immunoblot analysis of p16 and the three members of the pRb protein family (pRb, pRb2/p130 and p107) were also performed; no change in the phosphorylation status of these proteins was recorded and only minor variations were detected in their levels, while no clear trend in response to increasing concentrations of the metal could be evidenced (data not shown).

### Effects of cadmium on prostate cancer cell lines

**Figure 2:**
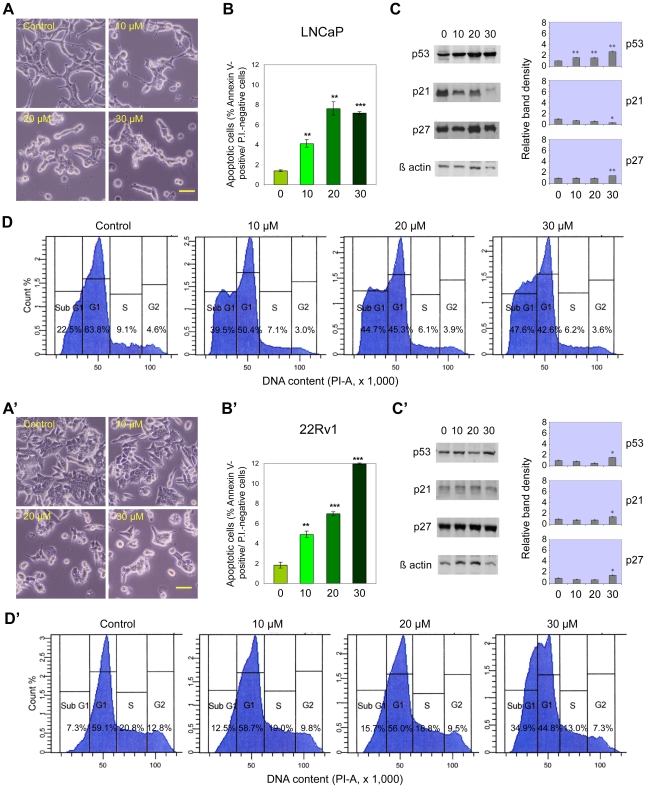
Cadmium induces dose-dependent apoptosis in LNCaP and 22Rv1 prostate cancer cell lines. Cadmium effects on LNCaP (A–D) and 22Rv1 (A′–D′) cells, after 24-h treatment with different concentrations of CdCl_2_ (0, 10, 20 and 30 µM). Same conditions as in figure 1. A clear correlation between apoptosis induction and p53 overexpression is particularly evident in wt p53-expressing LNCaP cells. Scale bars in A, A′ = 100 µm. * P<0.05, ** P<0.01, *** P<0.001.

**Figure 3:**
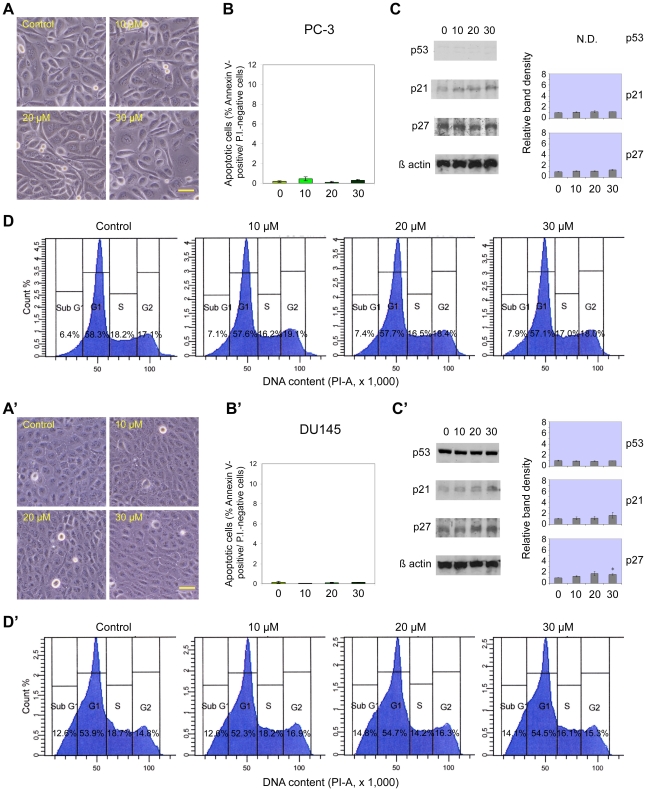
Cadmium does not induce apoptosis in p53-deficient PC3 and DU145 prostate cancer cell lines. Cadmium effects on PC3 (A–D) and DU145 (A′–D′) cells, after 24-h treatment with different concentrations of CdCl_2_ (0, 10, 20 and 30 µM). Same conditions as in figure 1. Scale bars in A, A′: 100 µm. * P<0.05.

The primary carcinoma 22Rv1 and the metastatic LNCaP cell lines responded in a similar way to 24-hour cadmium treatment, both exhibiting, in a dose-dependent manner and starting from 10 µM cadmium chloride, apoptosis induction (Fig. 2A, B and A′, B′), increase in the proportion of sub-G1 cells and reduction in S+G2/M fraction, as well as decreased G1 fraction (Fig. 2D, D′), clearly visible in 22Rv1 cells only at 30 µM. On the other hand, the other primary carcinoma cell line, CWR-R1, did not display any significant effects at cadmium doses lower than 30 µM: only at this concentration a significant increase in apoptosis and in subG1 cells could be found, together with a decrease in S+G2/M cell fraction (not shown). Differently from all these cell lines, the metastatic prostate cancer PC-3 and DU145 cells were not affected by cadmium toxic activity in the range of concentrations employed (Fig. 3A, B, D and A′, B′ and D′).

As regards the expression of tumor suppressor proteins, only LNCaP cells exhibited a significant concentration-dependent increase in total p53 after cadmium exposure (Fig. 2C′). A relatively small, 1.6-fold increase could also be detected in 22Rv1 cells at 30 µM cadmium (p<0.05; Fig. 2C). Conversely, no variations in p53 levels were observed in CWR-R1 (not shown) and DU145 (Fig. 3C′) cell lines, displaying high basal protein expression, whereas PC-3 cells did not express detectable amounts of p53 protein (Fig. 3C). Accordingly, the levels of p21 followed a similar pattern of expression, with a 1.4-fold increment in 22Rv1 at 30 µM cadmium (p<0.05; Fig. 2C), while they remained essentially unchanged in CWR-R1 (not shown), and PC-3 and DU145 cell lines (Fig. 3C, C′). On the contrary, and unexpectedly, in LNCaP cells p21 levels seemed to decrease with increasing cadmium chloride concentrations (Fig. 2C′). Regarding p27 levels, no significant variations were observed after 24-hour cadmium treatment in PC-3 (Fig. 3C) and CWR-R1 (not shown) cells, at any of the tested cadmium concentrations. On the other hand, 22Rv1, LNCaP and DU145 cell lines displayed slightly increased (about 1.5-fold) levels of p27 only at the highest concentration employed (30 µM; Fig. 2C, C′ and Fig. 3C′).

### Effects of siRNA-induced silencing of p53 protein in prostate epithelial cells

**Figure 4:**
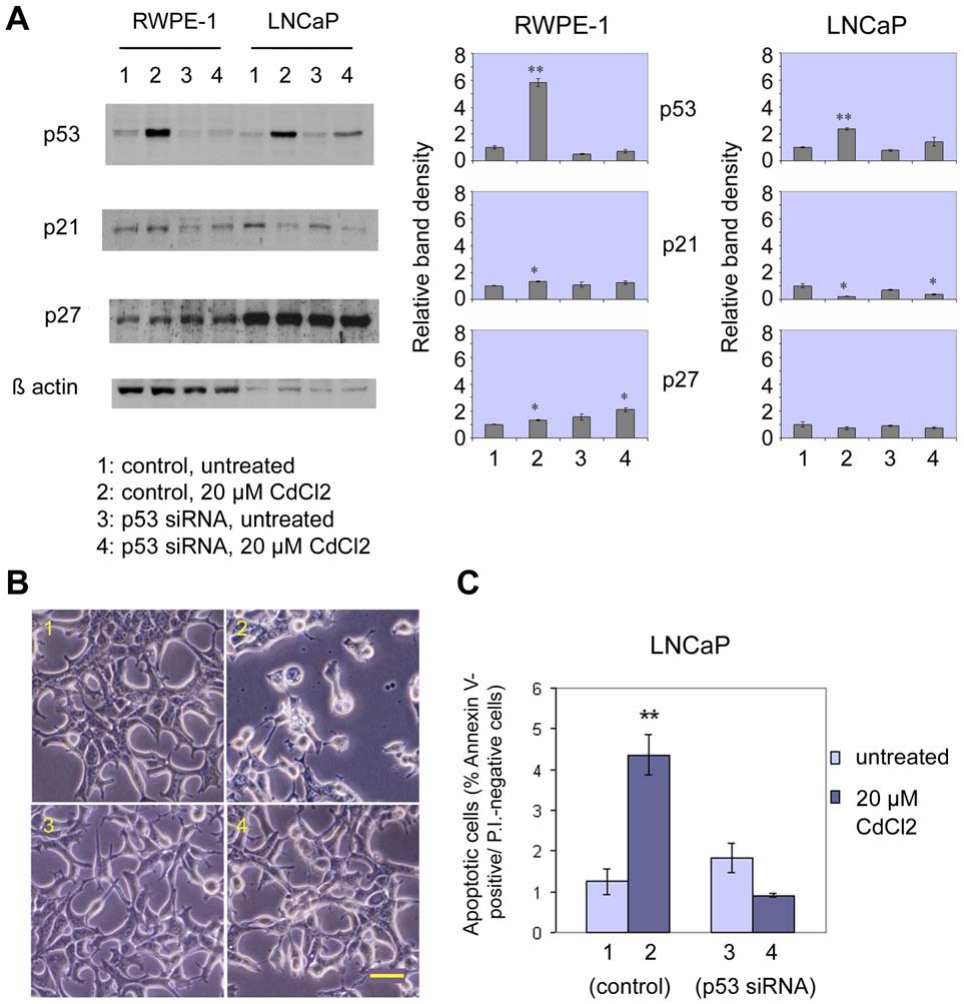
Silencing of p53 suppresses apoptosis induction by cadmium in human prostate cells. p53 siRNA transfection: western blot analysis (RWPE-1 and LNCaP cells) and early apoptosis detection (LNCaP cells). WB analysis of p53, p21 and p27 expression (A), representative phase contrast microscopy images (B) and FITC-conjugated Annexin-V/PI and FACS analysis (C; histograms, reporting mean percentages ± SEM of Annexin-V positive/PI-negative cells, n = 3) after p53 siRNA transfection, followed by 24-h treatment with 20 µM CdCl_2_. In LNCaP cells siRNA-mediated p53 silencing is able to suppress apoptosis induction by 24-hour exposure to 20 µM cadmium chloride, as compared to the respective control. WB histograms represent relative band densities (mean ± SEM, n = 3), as determined by densitometry, using β-actin as the loading control for standardization. Scale bar in B: 100 µm. *P<0.05, **P<0.01.

Since western blot analysis clearly revealed that cadmium apoptosis induction is correlated with elevations in p53 levels, at least in cell lines expressing wild-type p53, silencing of p53 by means of specific small interfering RNA was attempted in order to verify the role played by this oncosuppressor in the response of RWPE-1 (normal) and LNCaP (metastatic) cells to cadmium exposure. After determining optimal experimental conditions for obtaining efficient transfection, RWPE-1 and LNCaP cells, both possessing normal, wild type *p53* genes, were transfected with p53-siRNA and, 2 days later, treated with 20 µM cadmium chloride. Then, apoptosis detection and western blot analysis were performed. Results of western blotting analysis (Fig. 4A) proved that effective silencing of p53 was obtained in both cell lines. This effect was particularly evident in p53-siRNA transfected cells that were treated with 20 µM cadmium chloride, since in these cells only a slight, non statistically significant increase in p53 levels was obtained, as compared to control cadmium-exposed cells, in which p53 levels were 6-fold (RWPE-1) or 2.4-fold (LNCaP) higher than in their respective untreated controls. Moreover, p21 levels changed in accordance with p53 variations in RWPE-1 cells. In LNCaP cell line, on the other hand, the protein levels of p21 were decreased after incubation with 20 µM cadmium chloride, both in control and in p53-siRNA transfected cells (p<0.05). This observation confirmed our previous results (Figure 2D′), and was a further indication that p21 regulation in LNCaP might be, at least in part, independent of p53. Conversely, no significant changes were found in the levels of p27 protein in LNCaP cells, as observed at the same concentration in previous experiments. In RWPE-1 cells, on the contrary, p27 levels were enhanced in both control and p53-siRNA transfected cells, after treatment with 20 µM cadmium chloride. In correlation with the effective p53 silencing no significant induction of apoptotic cell death was observed in p53-siRNA transfected cells after 24-hour exposure to 20 µM cadmium chloride, as compared to the relative control. This was clearly evident in LNCaP cells (Fig. 4B,C). In the case of RWPE-1, unfortunately p53-siRNA transfection caused a small increase in the basal level of early apoptosis, which is indicative of a certain sensitivity of these cells to the possible toxic consequences of p53-siRNA treatment per se (data not shown).

### Effects of the overexpression of wild type p53 in p53-deficient prostate cancer cell lines

To further verify whether prostate epithelial cell sensitivity to cadmium is dependent on p53, the effects of restoration of wild type p53 expression in p53-defective DU145 and PC-3 cell lines were investigated. PC-3 are p53-null, while DU145 cells carry two mutations (codons 223 and 274) in the *p53* gene (Table 1) and express a non-functional mutant form of the protein, which tends to accumulate intracellularly. Overexpression of wt p53 was obtained by means of transduction with a recombinant Adp53 adenoviral vector. Subsequently, transduced cells were treated for 24 hours with different concentrations of cadmium chloride (in the range 0–30 µM) and were tested for apoptosis induction, cell cycle distribution and expression of p53 and p21 tumor suppressor proteins.

**Figure 5:**
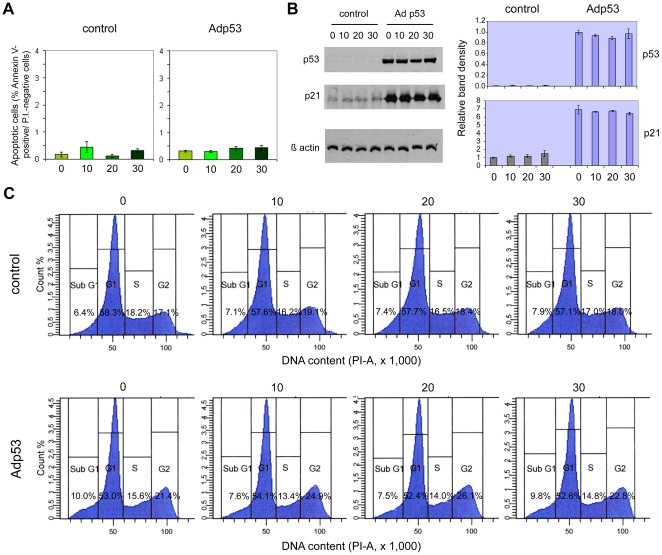
Effects of wt p53 overexpression and cadmium treatment in PC-3 cells. A: early apoptosis detection. The frequencies of apoptotic cells were determined in both Adp53-transduced and control PC-3 cells by FITC-conjugated Annexin-V/PI and FACS analysis after 24-h treatment with different CdCl_2_ concentrations (0,10, 20 and 30 µM). Histograms represent mean percentages ± SEM of Annexin-V positive/PI-negative cells (n = 3). B: western blot analysis. The levels of total p53 and p21 proteins were examined after Adp53-mediated overexpression of wild type p53, followed by 24-h treatment with different CdCl_2_ concentrations (0,10,20 and 30 µM). Histograms represent relative band densities (mean ± SEM, n = 3), as determined by densitometry, using β-actin as the loading control for standardization. C: cell cycle distribution. FACS analysis of Adp53-transduced and control PC-3 cells was performed after 24-h treatment with different CdCl_2_ concentrations (0, 10, 20 and 30 µM).

**Figure 6:**
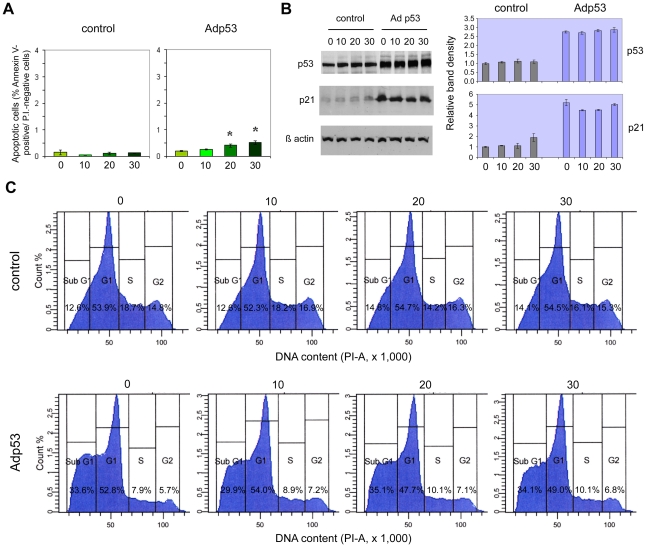
Effects of wt p53 overexpression and cadmium treatment in DU145 cells. Early apoptosis detection (A), western blot analysis of p53 and p21 proteins (B) and cell cycle distribution (C) in Adp53-transduced and control DU145 cells after 24-h treatment with different CdCl_2_ concentrations (0, 10, 20 and 30 µM). Same conditions as in figure 5.

In the case of PC-3, western blot analysis (Fig. 5B) demonstrated the successful expression of high levels of wt p53 protein in these p53-null cells, with the concomitant highly enhanced (about 7-fold) expression of the p53 target gene *p21*, as compared to non-transduced cells. The levels of the p21 protein remained, however, unchanged after cadmium chloride treatment. In accordance with the increased expression of p21, cell cycle distribution was, to a certain extent, altered in comparison with non-transfected cells: in particular, a higher proportion of cells in the G2/M fraction was found, together with reduced percentages of cells in the G1 and S phases and a minor increase in the sub-G1 fraction (Fig. 5C). But, again, 24-hour exposure to cadmium chloride was unable to induce any significant changes in the relative proportions of the different cell cycle fractions. As regards apoptosis detection, no significant differences were seen between Adp53-transfected and non-transfected cells, and no variations occurred following cadmium chloride treatment (Fig. 5A). Partly similar results were obtained with DU145 cells. Adp53 transfection caused wt p53 overexpression and a parallel elevation (about 5-fold) in the expression levels of p21 (Fig. 6B). As in PC-3, p21 levels were substantially unaffected by cadmium chloride treatment. Moreover, Adp53 overexpression caused a change in the cell cycle distribution, but different from what observed in PC-3 cells: the main effects in DU145 cells were a marked reduction in the proportion of cells in the S+G2/M fraction and a striking increase in the sub-G1 fraction (Fig. 6C). Also in this cell line, however, cadmium treatment did not seem to cause any variations in the cell cycle with respect to the control. The only, minimal effect obtained after Adp53 overexpression in DU145 cells was a slightly increased susceptibility to the induction of apoptosis by cadmium chloride, which was significantly different from control cells (p<0.05) at 20 and 30 µM concentrations (Fig. 6A), although the detected levels of early apoptotic cells were anyhow very low.

## Discussion

An established lung carcinogen in humans [4], the heavy metal cadmium is also suspected of playing a role in the induction and the development of prostate cancer in exposed subjects. A relation between cadmium exposure and prostate cancer etiology seems to be supported by laboratory and epidemiologic studies [3], but the specific molecular events associated with cadmium-induced transformation are still elusive. As reported above, different possible mechanisms may be involved in cadmium carcinogenesis. A key role, in particular, has been proposed for apoptosis induction and the possible subsequent acquisition of apoptotic resistance, as already observed during malignant transformation of cadmium-exposed and -adapted cells [21], [22]. Our investigation was aimed at characterizing the cadmium-induced apoptotic response in normal and tumor cells derived from human prostate epithelium at doses relevant to human exposure. In this regard, we used cadmium concentrations (10–30 µM) that are within the range that have been found in normal, hypertrophic and malignant human prostate tissues [26] and that, at the same time, are able to trigger apoptosis in cell culture systems [27]. Higher cadmium concentrations are more prone to cause necrosis [27] and were thus beyond the scope of our study.

As expected, normal and cancer prostate cells have exhibited rather dissimilar response after 24-hour cadmium treatment. If we consider both apoptosis induction and inhibition of cell proliferation the normal RWPE-1 cell line resulted, on the whole and with the exception of LNCaP cells, the most sensitive to cadmium cytotoxicity. On the other hand, its cadmium-transformed derivative CTPE, proved to be more resistant to the metal toxicity. Differences in results have been even conspicuous among prostate cancer cells of different origin. LNCaP cells (derived from a lymph node metastasis, androgen-responsive and relatively androgen-dependent, see [28]) exhibited a very high sensitivity to cadmium cytotoxic action. Of the remaining cell lines, the primary carcinoma-derived 22Rv1 and CWR-R1, resulted more sensitive than the two metastatic cell lines, DU145 and PC-3. These latter were not significantly affected by cadmium treatment in the range of concentrations employed.

Interestingly, cadmium sensitivity and apoptosis induction in prostate cells clearly correlated to the expression of functional, wild-type p53 protein. In fact, like the normal RWPE-1 cell line, also LNCaP cells, that resulted the most cadmium-sensitive prostate cancer cell line in our experiments, express wt p53. Conversely, all the other prostate cancer lines (22Rv1, CWR-R1, DU145 and PC-3) have mutated versions of the protein (Table 1, [29]–[35]). CTPE cells express wt p53 as well, but they are intrinsically more resistant to cadmium as a result of a specific selection procedure [12]; these cadmium-transformed cells, for instance, display a Bcl-2/Bax ratio about 5 times higher than in the parental RWPE-1 cell line, which confers them a higher resistance to apoptosis induction [21]. Western blot analysis confirmed this correlation between wt p53 expression and cadmium-induced apoptosis in prostate cells, revealing a strong, concentration-dependent increase in p53 levels in both RWPE-1 and LNCaP cells exposed to cadmium, parallel to the dose-dependent increase in apoptotic cell death. A similar, but less evident effect was observed also in CTPE cells, while a slight response was obtained at the highest Cd-concentration also in the p53-mutated 22Rv1 cells. This could be explained by the observation that 22Rv1 cells, although carrying a mutated *p53* allele, might retain the ability to synthesize a small quantity of functional p53 protein, since they still contain a wt *p53* allele [25].

Indeed, a major role in cadmium-induced apoptosis has already been suggested for the p53 tumor suppressor protein. Increased levels of p53 protein and mRNA have been observed in different cell lines undergoing apoptotic cell death after cadmium treatment, including primary epithelial lung cells [36], skin epidermal cells [37] and embryonic fibroblasts [38]. In agreement with our results, Achanzar et al. reported increased expression of p53, together with c-myc and c-jun, in the same normal prostate cell line (RWPE-1) that has been used here, after exposure to 10 µM cadmium chloride [20]. In particular, they found transiently increased levels of *p53* mRNA, as a prelude to subsequent massive apoptosis induction. Similar results (increased *p53* mRNA levels and consequent apoptosis) were also obtained by Zhou et al. in the ventral prostate of rats, after a single subcutaneous injection of cadmium chloride solution [39].

In addition, our analysis of p21 expression in cadmium-treated cells has indirectly confirmed the observed variations in p53 levels. A member of the *Kip/Cip*-family of CDK-inhibitors, *p21* (also known as *p21^Cip1/Waf1^*) is a p53-downstream gene that mediates p53-induced growth arrest in G1, mainly by suppressing the activity of the cyclin A, E/CDK2 complex, necessary for G1/S transition [40]. As the product of a target gene of p53, p21 levels may serve as an indirect indicator of the activation state of p53 in the cell. Looking at our results, it is evident that p21 levels follow a pattern of expression largely comparable to that of p53, in the normal RWPE-1 cell line, as well as in CTPE and 22Rv1 cells, indicating that the observed, dose-dependent increases in the levels of p53 after cadmium exposure are really corresponding to its activation, as a response to toxic stress. On the other hand, intriguingly, p21 protein levels tend to decrease while p53 is increasing in LNCaP cells; an apparently paradoxical phenomenon that has been, however, already observed by Tang et al. who reported, in LNCaP cells, time-dependent increase in p53 levels upon growth factor withdrawal, with consequent apoptosis induction, and concomitant gradual decrease in the levels of p21 [41]. In this regard, the authors concluded that: (a) p21 expression may be unrelated to or independent of p53 status, as suggested by other data; and (b) p53 and p21 may be involved in prostate cell apoptosis independently of each other, as observed in oncogene-mediated fibroblast apoptosis.

A further confirmation of the key role played by p53 in prostate cell apoptosis has come from the results of our experiments of siRNA-induced silencing, at least in LNCaP cells, in which the knockdown of p53 protein levels by p53siRNA transfection has been sufficient to reduce the sensitivity to 20 µM cadmium chloride, an otherwise highly toxic dose to these cells.

What signaling pathways are involved in p53 activation following cadmium exposure of prostate epithelial cells? Considering its role of “guardian of the genome”, acting as a key checkpoint of cellular behaviour in response to DNA damage [42], one could expect p53 to be activated by cadmium-induced genotoxic stress, through ATM/ATR protein kinases-mediated uncoupling of p53 from its main negative regulators MDM2 and MDM4, leading to accumulation of stable active p53 protein [43]. In reality, as already mentioned, cadmium has very little, if any, direct genotoxic activity at doses comparable with those used in the present study [6], even if it seems to be able to inhibit DNA repair and act as a co-genotoxic agent [17]–[19]. Moreover, preliminary experiments we performed to assess cadmium genotoxicity on prostate cells by means of cytokinesis-block micronucleus assay, failed to reveal any significant genotoxic effects (Text S1 and Table S1). If confirmed, these last results would indicate that some other type of cellular stress is responsible for p53 activation in cadmium-exposed prostate cells. Particularly interesting in this regard seems to be the role played by oncogenic signaling, because one of the reported effects of cadmium, obtained in different cell types, is the activation of proto-oncogenes, including *c-fos*, *c-myc* and *c-jun*, [15], [16], [44], [45]. This effect has been described also in the normal prostate RWPE-1 cell line, in which induction of *c-myc* and *c-jun* concomitant with p53 up-regulation was observed after treatment with 10 µM cadmium chloride [20]. Therefore, it is not unlikely that cadmium-induced p53 up-regulation in prostate cells may be largely due to proto-oncogene activation, leading to ARF induction, with the consequent inhibition of MDM2 independently of the DNA damage pathway [43]. The possibility of an alternative explanation, however, is suggested by some recently published results. In particular, it has been found that cadmium exposure of rat renal tubular cells can cause down-regulation of ubiquitin conjugating enzymes of the Ube2d family with increased levels of p53. Considering that some Ube2d family members seem to be involved in p53 ubiquitination and degradation, these results would indicate that cadmium-induced p53 accumulation could also be due to inhibition of its degradation through suppression of *Ube2d* gene expression [46].

In apparent contrast with these data, our results of wt p53 overexpression in p53-deficient DU145 and PC-3 cell lines would not seem to support a role for this protein in cadmium-induced apoptosis. Despite high levels of wt p53 were expressed in both cell lines after Adp53 transduction, with concomitant net increase in p21 levels and reduced proliferation, no effects on apoptosis susceptibility after cadmium exposure were seen in PC-3 cells, and only minimal effects were found in DU 145 cells. In these metastatic cancer lines, p53 functional restoration seems insufficient to enhance the sensitivity to this toxic metal, but this does not necessarily rule out a crucial function of p53 in triggering apoptosis by cadmium. Instead, it may simply indicate that other factors, besides lack of functional p53, may be responsible for PC-3 and DU145 resistance to cadmium-induced apoptosis. One such factor may be represented by metallothioneins (MT), metal-binding proteins whose basal or induced levels may influence the susceptibility to cadmium toxic effects, including apoptosis [47]. Yet, induction of MT-I by cadmium was reported in the cadmium-sensitive LNCaP cell line, while, on the contrary, the MT-I promoter was found suppressed in the cadmium-resistant DU145 and PC-3 cells [48], which suggests that MTs should not be determinant for the observed differences in the response of our prostate cell lines. An alternative explanation might be in the different expression of other regulators of the apoptotic pathway(s), and particularly the antiapoptotic proteins called IAPs (Inhibitors of Apoptosis Proteins); in fact, McEleny et al. reported that PC-3 and DU145 cells express higher mRNA and/or protein levels of numerous IAPs, as compared to the LNCaP cell line, which may well be associated with the observed differences in resistance to apoptosis, even after p53 functional restoration [49].

As regards p27, it is noteworthy that this key player in cell cycle regulation has shown only minimal effects in the presence of cadmium. Reduced cellular levels of p27, mostly due to deregulation of the post-translational processing of the protein, have been associated to malignant progression [50]. In particular, low levels or loss of p27 have been shown to correlate with more aggressive phenotype and poor survival in prostate cancer [51], [52]. Nevertheless, clearly detectable amounts of p27 (often relatively high, such as in 22Rv1 and LNCaP) have always been found in all the prostate cell lines used here, even if it can not be excluded that one or more of them express mutated p27. The general lack of a significant increase of this protein after cadmium treatment at concentrations <30 µM suggests, rather, that cadmium at low doses is not able to induce p27 up-regulation and the consequent p27-mediated growth inhibition in these prostate cell lines. Instead, in cadmium-responsive cells (more specifically RWPE-1, LNCaP, CTPE and, to a minor extent, 22Rv1 cells), the effects of low-dose cadmium on cell survival, apoptosis-induction and cell cycle regulation seem to be mostly mediated by the activation of p53 and its downstream target p21 (with the possible exception, for this latter, of LNCaP cells).

In conclusion, the results presented here have revealed that cadmium has the ability to induce p53-dependent apoptotic cell death in human prostate epithelial cells. As demonstrated by our comparative analysis, wild type p53-expressing cell lines are more sensitive to cadmium treatment than cells harboring mutated *p53* alleles, while apoptosis induction and inhibition of cell proliferation positively correlate with accumulation of functional p53 protein and overexpression of the cell cycle inhibitor p21, a well characterized p53 target (except in the case of LNCaP cells, apparently exhibiting a peculiar inverse relationship between p53 and p21 expression). We have further assessed this p53 dependence in a more direct manner, by using a p53 siRNA strategy to prevent accumulation of p53 protein in RWPE-1 and LNCaP cells following cadmium chloride exposure. Conversely, adenoviral p53 gene transfer was not sufficient to “restore” cadmium sensitivity in PC-3 and DU145 cells, which are not able to express functional p53 protein. This last finding implies that PC-3 and DU145 resistance to cadmium may depend not only on the absence of wild type *p53* gene, but also on some other mechanism(s) like, for instance, the overexpression of IAPs.

Together, our results identify p53 as a major player in cadmium-induced apoptosis in the prostate. Furthermore, by showing that prostate cells carrying mutated *p53* alleles are able to escape from apoptosis induction, they suggest a possible selection mechanism operating in prostate cell populations exposed to cadmium toxic action, through which p53-mutated cells, that are more prone to give rise to tumor formation, may survive and become predominant, while wt p53 cells are progressively removed by apoptosis. Since the acquisition of apoptotic resistance may be crucial in cadmium-induced malignant transformation, as already evidenced in cadmium-adapted cells displaying a significantly attenuated apoptotic response, further characterization of the pathways involved and a thorough comparative proteome analysis of the different prostate epithelial cells studied here, treated or not with this heavy metal, would certainly improve our understanding of cadmium carcinogenesis in the prostate.

## Supporting Information

Table S1 **Absence of micronucleus induction in different prostate epithelial cell lines, after 2-day exposure to 3 or 10 µM CdCl_2_.** The percentages of binucleated cells with micronuclei are reported in the column evidenced in purple. Examples of binucleated cells with micronuclei, one for each cell line, are shown in the images on the right. * P<0.05, ** P<0.01.
(TIF)

Text S1 **Evaluation of cadmium genotoxicity in prostate epithelial cell lines by means of cytokinesis-block micronucleus (CBMN) assay.**
(DOC)

## References

[1] Siegel R, Ward E, Brawley O, Jemal A (2011). Cancer Statistics, 2011. The impact of eliminating socioeconomic and racial disparities on premature cancer deaths.. CA Cancer J Clin.

[2] Crawford ED (2009). Understanding the Epidemiology, Natural History, and Key Pathways Involved in Prostate Cancer.. Urology.

[3] Huff J, Lunn RM, Waalkes MP, Tomatis L, Infante PF (2007). Cadmium-induced cancers in animals and in humans.. Int J Occup Environ Health.

[4] IARC (1993). Monographs on the Evaluation of Carcinogenic Risks to Humans, vol. 58, Beryllium, Cadmium, Mercury, and Exposures in the Glass Manufacturing Industry..

[5] National Toxicology Program (2000). Tenth Report on Carcinogens..

[6] Waalkes MP (2003). Cadmium carcinogenesis.. Mutat Res.

[7] Waalkes MP, Rehm S, Riggs CW, Bare RM, Devor DE (1988). Cadmium carcinogenesis in male Wistar [Crl:(WI)BR] rats: dose-response analysis of tumor induction in the prostate and testes and at the injection site.. Cancer Res.

[8] Waalkes MP, Rehm S, Riggs CW, Bare RM, Devor DE (1989). Cadmium carcinogenesis in male Wistar [Crl:(WI)BR] rats: dose-response analysis of effects of zinc on tumor induction in the prostate, in the testes, and at the injection site.. Cancer Res.

[9] Waalkes MP, Anver M, Diwan B (1999). Carcinogenic effects of cadmium in the Noble (NBL/Cr) Rat: induction of pituitary, testicular, and injection site tumors and intraepithelial proliferative lesions of the dorsolateral prostate.. Toxicol Sci.

[10] Waalkes MP, Anver M, Diwan B (1999). Chronic toxic and carcinogenic effects of oral cadmium in the Noble (NBL/Cr) Rat: induction of neoplastic and proliferative lesions of the adrenal, kidney, prostate and testes.. J Toxicol Environ Health.

[11] Terracio L, Nachtigal M (1988). Oncogenicity of rat prostate cells transformed in vitro with cadmium chloride.. Arch Toxicol.

[12] Achanzar WE, Diwan BA, Quader ST, Webber MM, Waalkes MP (2001). Cadmium-induced malignant transformation of human prostate epithelial cells.. Cancer Res.

[13] Nakamura K, Yasunaga Y, Ko D, Xu LL, Moul JW (2002). Cadmium-induced neoplastic transformation of human prostate epithelial cells.. Int J Oncol.

[14] Joseph P (2009). Mechanisms of cadmium carcinogenesis.. Toxicol Appl Pharmacol.

[15] Bertin G, Averbeck D (2006). Cadmium: cellular effects, modifications of biomolecules, modulation of DNA repair and genotoxic consequences (a review).. Biochimie.

[16] Beyersmann D, Hechtenberg S (1997). Cadmium, Gene Regulation, and Cellular Signaling in Mammalian Cells.. Toxicol Appl Pharmacol.

[17] Giaginis C, Gatzidou E, Theocharis S (2006). DNA repair systems as targets of cadmium toxicity.. Toxicol Appl Pharmacol.

[18] Hartwig A (2010). Mechanisms in cadmium-induced carcinogenicity: recent insights.. Biometals.

[19] Waisberg M, Joseph P, Hale B, Beyersmann D (2003). Molecular and cellular mechanisms of cadmium carcinogenesis.. Toxicology.

[20] Achanzar WE, Achanzar KB, Lewis JG, Webber MM, Waalkes MP (2000). Cadmium induces c-myc, p53, and c-jun expression in normal human prostate epithelial cells as a prelude to apoptosis.. Toxicol Appl Pharmacol.

[21] Achanzar WE, Webber MM, Waalkes MP (2002). Altered apoptotic gene expression and acquired apoptotic resistance in cadmium-transformed human prostate epithelial cell.. Prostate.

[22] Hart BA, Potts RJ, Watkin RD (2001). Cadmium adaptation in the lung—a double-edged sword?. Toxicology.

[23] Hanahan D, Weinberg RA (2000). The hallmarks of cancer.. Cell.

[24] Bello D, Webber MM, Kleinman HK, Wartinger DD, Rhim JS (1997). Androgen responsive adult human prostatic epithelial cell lines immortalized by human papillomavirus 18.. Carcinogenesis (Lond.).

[25] van Bokhoven A, Varella-Garcia M, Korch C, Johannes WU, Smith EE (2003). Molecular Characterization of Human Prostate Carcinoma Cell Lines.. Prostate.

[26] Ogunlewe JO, Osegbe DN (1989). Zinc and cadmium concentrations in indigenous blacks with normal, hypertrophic and malignant prostate.. Cancer.

[27] Templeton DM, Liu Y (2010). Multiple roles of cadmium in cell death and survival.. Chem Biol Interact.

[28] Horoszewicz JS, Leong SS, Chu TM, Wajsman ZL, Friedman M (1980). The LNCaP cell line- A new model for studies on human prostatic carcinoma.. Prog Clin Biol Res.

[29] Sramkoski RM, Pretlow TG, Giaconia JM, Pretlow TP, Schwartz S (1999). A new human prostate carcinoma cell line, 22Rv1.. In Vitro Cell Dev Biol Anim.

[30] Gregory CW, Johnson RT, Mohler JL, French FS, Wilson EM (2001). Androgen receptor stabilization in recurrent prostate cancer is associated with hypersensitivity to low androgen.. Cancer Res.

[31] Mickey DD, Stone KR, Wunderli H, Mickey JH, Vollmer RT (1977). Heterotransplantation of a human prostatic adenocarcinoma cell line in nude mice.. Cancer Res.

[32] Stone KR, Mickey DD, Wunderli H, Mickey JH, Paulson DF (1978). Isolation of a human prostate carcinoma cell line (DU 145).. Int J Cancer.

[33] Kaighn ME, Narayan KS, Ohnuki Y, Lechner JF, Jones LW (1979). Establishment and characterization of a human prostatic carcinoma cell line (PC-3).. Invest Urol.

[34] Isaacs WB, Carter BS, Ewing CM (1991). Wild-type p53 suppresses growth of human prostate cancer cells containing mutant p53 alleles.. Cancer Res.

[35] Carroll AG, Voeller HJ, Sugars L, Gelmann EP (1993). p53 oncogene mutations in three human prostate cancer cell lines.. Prostate.

[36] Lag M, Westly S, Lerstad T, Bjornsrud C, Refsnes M (2002). Cadmium-induced apoptosis of primary epithelial lung cells: involvement of Bax and p53, but not oxidative stress.. Cell Biol Toxicol.

[37] Son YO, Lee JC, Hitron JA, Pan J, Zhang Z (2010). Cadmium induces intracellular Ca^2+^ and H_2_O_2_-dependent apoptosis through JNK- and p53-mediated pathways in skin epidermal cell line.. Toxicol Sci.

[38] Yu X, Sidhu JS, Hong S, Robinson JF, Ponce RA (2011). Cadmium induced p53-dependent activation of stress signaling, accumulation of ubiquinated proteins, and apoptosis in mouse embryonic fibroblast cells.. Toxicol Sci.

[39] Zhou T, Zhou G, Song W, Eguchi N, Lu W (1999). Cadmium-induced apoptosis and changes in expression of p53, c-jun and MT-I genes in testes and ventral prostate of rats.. Toxicology.

[40] el Deiry WS, Tokino T, Velculescu VE, Levy DB, Parsons R (1993). WAF1, a potential mediator of p53 tumor suppression.. Cell.

[41] Tang DG, Li L, Chopra DP, Porter AT (1998). Extended Survivability of Prostate Cancer Cells in the Absence of Trophic Factors: Increased Proliferation, Evasion of Apoptosis, and the Role of Apoptosis Proteins.. Cancer Res.

[42] Vousden KH (2000). p53: death star.. Cell.

[43] Meek DW (2009). Tumour suppression by p53: a role for the DNA damage response?. Nat Rev Cancer.

[44] Joseph P, Muchnok TK, Klishis ML, Roberts JR, Antonini JM (2001). Cadmium-induced cell transformation and tumorigenesis are associated with transcriptional activation of c-fos, c-jun,and c-myc proto-oncogenes: role of cellular calcium and reactive oxygen species.. Toxicol Sci.

[45] Qu W, Diwan BA, Reece JM, Bortner CD, Pi J (2005). Cadmium-induced malignant transformation in rat liver cells: role of aberrant oncogene expression and minimal role of oxidative stress.. Int J Cancer.

[46] Tokumoto M, Fujiwara Y, Shimada A, Hasegawa T, Seko Y (2011). Cadmium toxicity is caused by accumulation of p53 through the down-regulation of *Ube2d* family genes *in vitro* and *in vivo*.. J Toxicol Sci.

[47] Pulido MD, Parrish AR (2003). Metal-induced apoptosis: mechanisms.. Mutat Res.

[48] Jacob ST, Majumder S, Ghoshal K (2002). Suppression of metallothionein-I/II expression and its probable molecular mechanisms.. Environ Health Perspect.

[49] McEleny KR, Watson RWG, Coffey RNT, O'Neill AJ, Fitzpatrick JM (2002). Inhibitors of apoptosis proteins in prostate cancer cell lines.. Prostate.

[50] Slingerland J, Pagano M (2000). Regulation of the cdk inhibitor p27 and its deregulation in cancer.. J Cell Physiol.

[51] Claudio PP, Zamparelli A, Garcia FU, Claudio L, Ammirati G (2002). Expression of cell-cycle-regulated proteins pRb2/p130, p107, p27(kip1), p53, mdm-2, and Ki-67 (MIB-1) in prostatic gland adenocarcinoma.. Clin Cancer Res.

[52] Guo Y, Sklar GN, Borkowski A, Kyprianou N (1997). Loss of the cyclin-dependent kinase inhibitor p27(Kip1) protein in human prostate cancer correlates with tumor grade.. Clin Cancer Res.

[53] van Bokhoven A, Varella-Garcia M, Korch C, Hessels D, Miller GJ (2001). Widely used prostate carcinoma cell lines share common origins.. Prostate.

