# Supplementary figures and images for: Cadmium Induces p53-Dependent Apoptosis in Human Prostate Epithelial Cells
Source: PLoS One. 2012 Mar 20;7(3):e33647. doi: 10.1371/journal.pone.0033647 (PMC3308998; doi:10.1371/journal.pone.0033647)

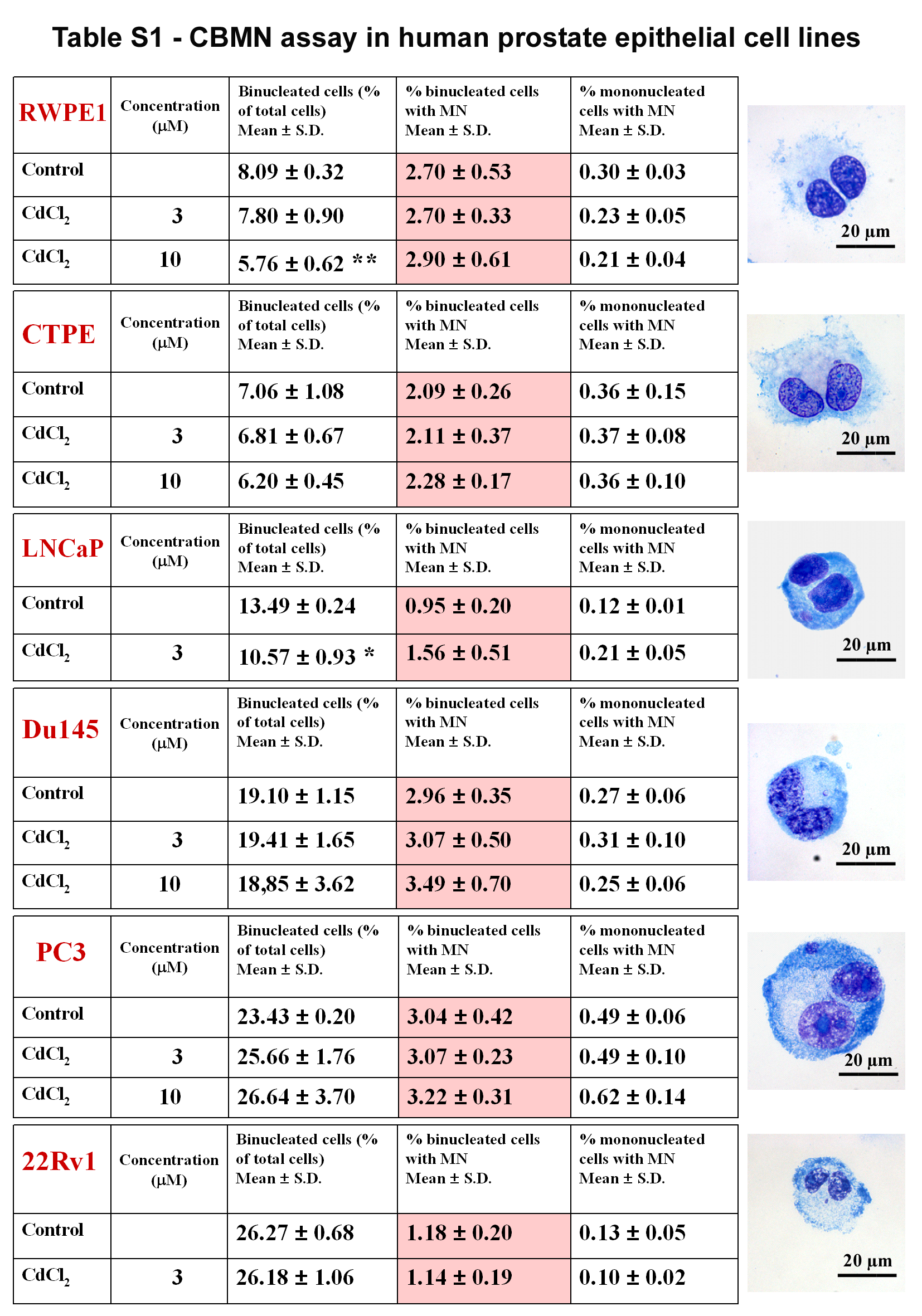

Supplement: Table S1 — Absence of micronucleus induction in different prostate epithelial cell lines, after 2-day exposure to 3 or 10 µM CdCl2. The percentages of binucleated cells with micronuclei are reported in the column evidenced in purple. Examples of binucleated cells with micronuclei, one for each cell line, are shown in the images on the right. * P<0.05, ** P<0.01. (TIF) [file pone.0033647.s001.tif]
