# Supplementary material for: Cadmium Induces p53-Dependent Apoptosis in Human Prostate Epithelial Cells
Source: PLoS One. 2012 Mar 20;7(3):e33647. doi: 10.1371/journal.pone.0033647 (PMC3308998; doi:10.1371/journal.pone.0033647)
Supplement: Text S1 — Evaluation of cadmium genotoxicity in prostate epithelial cell lines by means of cytokinesis-block micronucleus (CBMN) assay. (DOC) [file pone.0033647.s002.doc]

**Supporting information, Text S1**

**Evaluation of cadmium genotoxicity in prostate epithelial cell lines by means of cytokinesis-block micronucleus (CBMN) assay**

To assess the ability of cadmium to induce genotoxic effects in prostate epithelial cells, RWPE-1, CTPE, 22Rv1, LNCaP, DU145 and PC3 cell lines were exposed for 2 days to cadmium chloride, and then subjected to cytokinesis-block micronucleus (CBMN) assay. CBMN method is recognized as a reliable genotoxicity test, particularly suitable for chromosome damage measurement, allowing at the same time the detection of both chromosome loss and breakage [1]. Two different concentrations of the metal compound were used, 3 and 10µM, both of which are sub-cytotoxic for these cells (except for 10µM in LNCaP and 22Rv1 cell lines, that were, in fact, tested only at 3µM) and comparable to cadmium levels measured in human prostate.

**Description of the method**

For the CBMN assay, cytochalasin-B was added to treated and control cultures at a final concentration of 3 μg/ml. Cultured cells were harvested 24 h later, washed in PBS, resuspended at a final concentration of about 5X10^6 cells per ml and spread onto glass slides (20μl of cell suspension per slide); after air-drying the cells were fixed with methanol/glacial acetic acid (3:1) for 10 min, and stained with 5 % Giemsa solution for 5 min. All the above treatments were conducted at room temperature. After washing with distilled water, slides were rapidly dried in xylene and mounted with Canada balsam. Observations were performed with a Leitz Ortholux light microscope at 400x or 1,000x magnification, and the presence of micronuclei in binucleated cells was evaluated according to the scoring criteria described by Fenech M. *et al.* [2]. The frequency of micronuclei (MN) was determined by analysing 1X10^3 binucleated cells from each coded slide, in triplicate for each condition, and the significance of the differences between treatments and respective controls was determined by Student’s *t* test. *P* < 0.05 was considered statistically significant.

**Results**

With no exceptions, 2-day treatment with either 3 or 10µM cadmium chloride did not induce any significant changes in the observed percentages of binucleated cells with micronuclei, in any of the employed cell lines (Table S1).

**References**

1. Fenech M (2000) The in vitro micronucleus technique. Mutat Res 455: 81-95.
2. Fenech M, Chang WP, Kirsch-Volders M, Holland N, Bonassi S, et al. (2003) HUMN project: detailed description of the scoring criteria for the cytokinesis-block micronucleus assay using isolated human lymphocyte cultures. Mutat Res 534: 65-75.
